# The effect of dapagliflozin treatment on epicardial adipose tissue volume

**DOI:** 10.1186/s12933-017-0658-8

**Published:** 2018-01-04

**Authors:** Takao Sato, Yoshifusa Aizawa, Sho Yuasa, Shohei Kishi, Koichi Fuse, Satoshi Fujita, Yoshio Ikeda, Hitoshi Kitazawa, Minoru Takahashi, Masahito Sato, Masaaki Okabe

**Affiliations:** Cardiology, Tachikawa General Hospital, 561-1 Jyojyomachi Aza Yauchi, Nagaoka, Japan

**Keywords:** SGLT-2 inhibitor, Epicardial adipose tissue, Diabetes mellitus

## Abstract

**Background:**

Glycosuria produced by sodium–glucose co-transporter-2 (SGLT-2) inhibitors is associated with weight loss. SGLT-2 inhibitors reportedly might reduce the occurrence of cardiovascular events. Epicardial adipose tissue (EAT) is a pathogenic fat depot that may be associated with coronary atherosclerosis. The present study evaluated the relationship between an SGLT-2 inhibitor (dapagliflozin) and EAT volume.

**Methods:**

In 40 diabetes mellitus patients with coronary artery disease (10 women and 30 men; mean age of all 40 patients was 67.2 ± 5.4 years), EAT volume was compared prospectively between the dapagliflozin treatment group (DG; n = 20) and conventional treatment group (CTG; n = 20) during a 6-month period. EAT was defined as any pixel that had computed tomography attenuation of − 150 to − 30 Hounsfield units within the pericardial sac. Metabolic parameters, including HbA1c, tumor necrotic factor-α (TNF-α), and plasminogen activator inhibitor-1 (PAI-1) levels, were measured at both baseline and 6-months thereafter.

**Results:**

There were no significant differences at baseline of EAT volume and HbA1c, PAI-1, and TNF-α levels between the two treatment groups. After a 6-month follow-up, the change in HbA1c levels in the DG decreased significantly from 7.2 to 6.8%, while body weight decreased significantly in the DG compared with the CTG (− 2.9 ± 3.4 vs. 0.2 ± 2.4 kg, p = 0.01). At the 6-month follow-up, serum PAI-1 levels tended to decline in the DG. In addition, the change in the TNF-α level in the DG was significantly greater than that in the CTG (− 0.5 ± 0.7 vs. 0.03 ± 0.3 pg/ml, p = 0.03). Furthermore, EAT volume significantly decreased in the DG at the 6-month follow-up compared with the CTG (− 16.4 ± 8.3 vs. 4.7 ± 8.8 cm^3^, p = 0.01). Not only the changes in the EAT volume and body weight, but also those in the EAT volume and TNF-α level, showed significantly positive correlation.

**Conclusion:**

Treatment with dapagliflozin might improve systemic metabolic parameters and decrease the EAT volume in diabetes mellitus patients, possibly contributing to risk reduction in cardiovascular events.

## Introduction

Obesity and diabetes mellitus (DM) are fast growing public health problems in industrialized countries [1] and are associated with cardiovascular complications [2]. Adipose tissue secretes many biologically active substances (or adipocytokines) including plasminogen activator inhibitor-1 (PAI-1) and tumor necrosis factor-α (TNF-α) [3]. In addition, serum levels of circulating pro-inflammatory cytokines are increased in overweight people with enhanced accumulation of visceral fat, leading to insulin resistance [4–6].

Pericardial fat is composed of epicardial fat (the epicardial adipose tissue (EAT) depot immediately adjacent to the heart wall) and pericardial fat located on the external surface of the pericardium (i.e., mediastinal fat) [7]. Fat depots localized around the heart (i.e., EAT) are involved in the pathogenesis of coronary artery disease [8]. EAT produces various bioactive molecules that significantly affect cardiac function [9]. Furthermore, EAT and pericardial fat volume are strongly correlated with each other and equally related to the number of atherosclerotic plaques [7].

Sodium–glucose co-transporter-2 (SGLT-2) inhibitors are novel oral hypoglycemic drugs that produce glycosuria by blocking the reabsorption glucose in renal proximal tubules. As a result, this mechanism is associated with body weight loss [10]. In addition, the administration of SGLT-2 inhibitors decreases abdominal visceral adipose deposits and improves nonalcoholic steatohepatitis [11, 12]. The EMPA-REG OUTCOME trial reported reduced death from cardiovascular causes, and decreased hospitalization for heart failure and progression to end-stage kidney disease, in patients with type 2 DM treated with SGLT-2 inhibitors, and established that there was a decreased risk of re-hospitalization for heart failure and the occurrence of myocardial infarction [13]. The present study was designed to investigate effects of an SGLT-2 inhibitor on EAT using multislice computed tomography (CT).

## Methods

### Study population

In 40 type-2 DM patients with coronary artery disease (CAD) (10 women and 30 men; mean age of all 40 patients was 67.2 ± 5.4 years), EAT volume was compared prospectively between the dapagliflozin and conventional treatment groups (both n = 20) during a 6-month period. Because there are few reports on the ability of SGLT-2 inhibitors to reduce EAT, we used a previous report on the effect of glucagon-like peptide-1 on EAT volume (approximately a 10% reduction) [14] to estimate a sample size of 17 subjects per group. Sample size estimates were based on the following: standard deviation, 10; α-level, 0.05; and power, 80%.

A total of 40 consecutive patients who received conventional medical therapy for CAD were recruited at the Tachikawa General Hospital. Patients were randomized into the dapagliflozin and conventional treatment groups. Group allocation was managed by an independent administrator. Thereafter, the addition of any medication was chosen. In the dapagliflozin group, only dapagliflozin was newly prescribed with medications before the allocation, while in the conventional treatment groups, oral hypoglycemic drugs except SGLT-2 inhibitors were newly prescribed with medications before the allocation. During the 6 months, the treatment regimen remained unchanged, except when unacceptable hyperglycemia, hypoglycemia, or adverse events occurred.

Patients who satisfied the following inclusion criteria were eligible for enrollment: [1] HbA1c level ≥ 6.5%, despite treatment with only diet and exercise therapy, or treatment with a combination of oral antidiabetic agents. Patients were excluded if they had age ≥ 75 years, insulin therapy, atrial fibrillation, iodine-based contrast agent allergy, renal insufficiency (estimated glomerular filtration rate < 45 mL/min/1.73 m^2^), chronic inflammatory disease, or coronary artery bypass graft surgery. Risk factors included were hypertension (oral treatment with antihypertensive drugs or documented history), hypercholesterolemia (oral treatment with lipid-lowering drugs or a total cholesterol measurement > 220 mg/dL), and cigarette smoking.

EAT volume was measured with multislice CT at baseline and 6 months after the randomization of patients. Similarly, blood samples were taken at baseline and 6 months later. The study protocol was approved by the institutional ethics committee of Tachikawa General Hospital, and written informed consent was obtained from all patients.

### Definition of CAD

Documented CAD encompassed one or more of the following: stable angina pectoris that was diagnosed with stress electrocardiology, or radioisotope or coronary angiography; previous acute coronary syndrome including unstable angina, and myocardial infarction that was diagnosed with coronary angiography and treated with percutaneous coronary intervention. However, the patients who underwent percutaneous coronary intervention for acute coronary syndrome, received optimal medical therapy and were enrolled 6 months after initial percutaneous coronary intervention.

### Laboratory tests

Blood samples were taken from the antecubital vein after a minimum of 12 h of fasting to determine serum levels of lipids [triglyceride (TG), high-density lipoprotein cholesterol (HDL), and low-density lipoprotein (LDL)], HbA1c [National Glycohemoglobin Standardization Program], insulin, and brain natriuretic peptide (BNP). In addition, samples of morning midstream urine were collected to evaluate the albumin/creatinine ratio. PAI-1 and TNF-α levels were measured at SRL (Tokyo, Japan), TNF-α was measured by an enzyme-linked immunosorbent assay using a commercial kit according to the manufacturer’s recommended protocol. Protein levels of total PAI-1 were determined using an LPIA-PAI test. Interactive, 24-variable model homeostatic model assessment insulin resistance-2 (iHOMA2%S) was measured as an index of insulin resistance based on a previous report [15]. In addition, HOMA-IR was calculated based on the following formula: HOMA-IR = [fasting serum insulin (μU/mL) × fasting blood sugar (mg/dL)/405].

### EAT volume measurement with multislice CT

For EAT measurements, CT angiography images were assessed using electrocardiography-gated cardiac CT scans on a 320-slice multi-detector computed tomography scanner (Aquilion One, Toshiba Medical Systems, Otawara, Japan). Subjects were examined in a supine position with their arms stretched above their heads. Nitroglycerin (0.3 mg) was administered to all subjects immediately before CT imaging. A β-blocker was also administered before CT imaging to ensure that the heart rates remained less than 65 beats/min, if required according to local institutional guidelines.

The coronary CT angiography protocol applied in the present study was as follows: slice collimation, 320 × 0.5 mm; gantry rotation time, 0.275 s; and tube voltage, 120 kV. At the time of scanning, a bolus of contrast medium (Omnipaque, 350 mg iodine/mL or iopamidol, 370 mg iodine/mL) was injected intravenously through an antecubital vein via an 18-gauge catheter, followed by 30 mL of normal saline. A site within the ascending aorta was selected as the region of interest (ROI), and the scan was initiated when the CT density reached the target level of 150 Hounsfield units more than the baseline density.

**Fig. 1:**
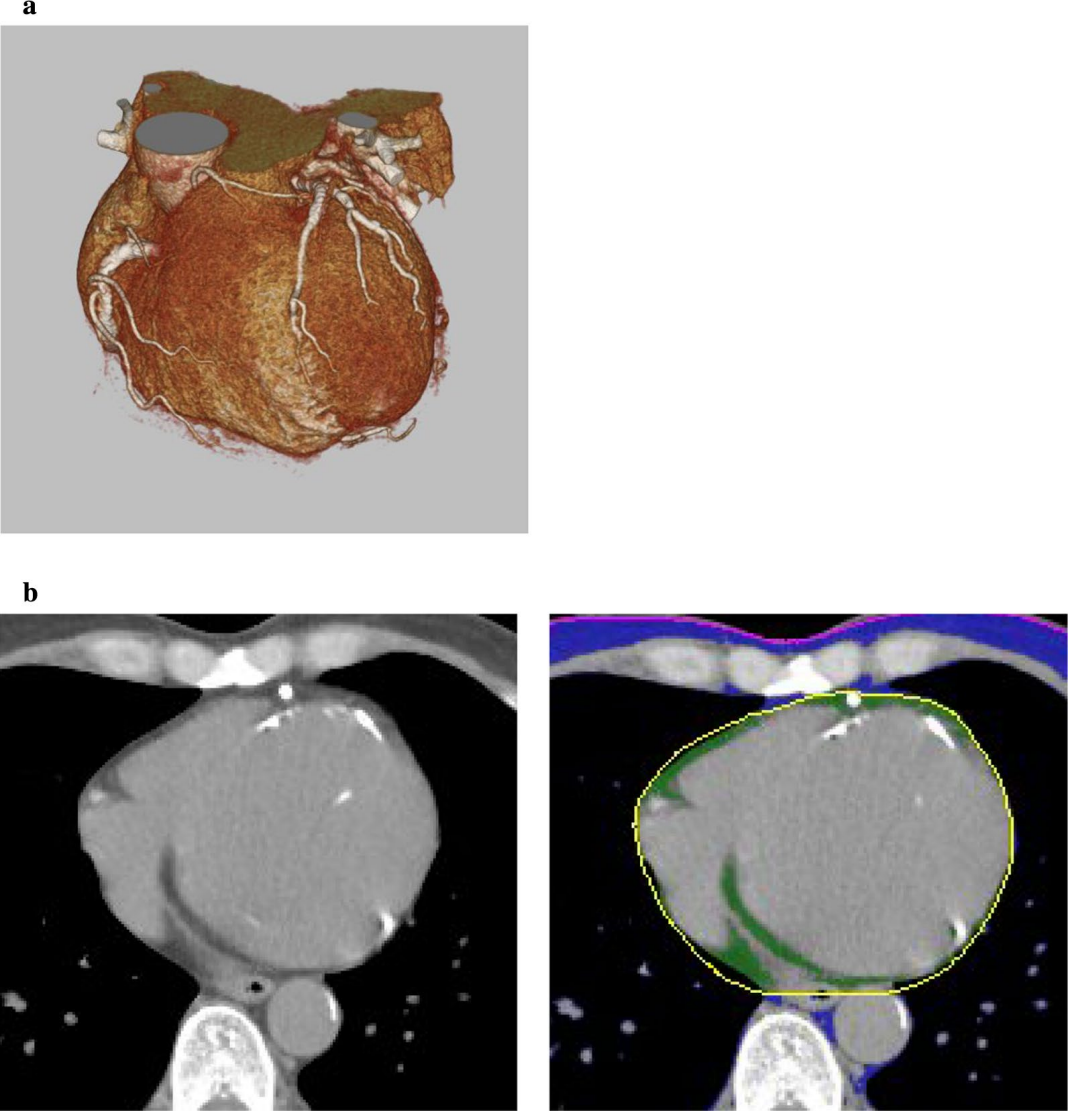
Measurement of epicardial adipose tissue (EAT) volumes with multislice CT. **a** 3D image. EAT area was measured on each axial image from the lower surface of left pulmonary artery origin to the left ventricular apex, and total EAT volume was obtained from multiplying total EAT areas by the slice thickness. **b** Axial images. A region of interest (ROI) was manually placed along the visceral pericardium (left and middle panel). EAT was extracted on an axial image (green) (right panel)

Volumetric measurements were performed on axial views with a 0.5-mm slice thickness. The superior border for measuring the EAT volume was set at the lower surface of the left pulmonary artery origin. The inferior border for the measurement was set at the left ventricular apex [16]. EAT area was calculated by tracing an ROI that included the heart and EAT. The ROI was manually placed outside the line of the visceral pericardium on the cross-sectional axial image. The area outside the traced pericardium was excluded (Fig. 1). EAT volumes were quantified by calculating the total volume of tissue having a CT density between − 150 and − 30 Hounsfield units (Fig. 1). EAT volume measurement by CT were performed by an experienced cardiologist who was blinded to the quantitative analysis data as well as baseline clinical characteristics.

### Statistical analyses

All statistical analyses were performed using SPSS version 22 (IBM Japan, Tokyo, Japan). Continuous data with a non-parametric statistical distribution are presented as mean ± standard deviation, and categorical data as count percentages. Comparisons between the dapagliflozin and conventional treatment groups were performed by Mann–Whitney’s U test for continuous data and Fisher’s exact test for categorical data. Furthermore, the correlations between the change in EAT volume and those in body weight or TNF-α level were evaluated using Spearman rank-order correlation coefficient analysis. A two-sided p-value of < 0.05 was considered statistically significant for all analyses.

## Results

### Patient characteristics

The overall mean age of the 40 patients was 67.2 ± 5.4 years. The average HbA1c value was 7.3 ± 0.3%. There were no significant differences in age, renal function, body weight, and medication use between the two groups at baseline (Table 1). Tables 2 and 3 shows the therapies for the patients in both groups.

**Table 1:** Baseline clinical characteristics

	Dapagliflozin (N = 20)	Conventional therapy (N = 20)	p value
Age (years)	68 ± 4	66 ± 6	0.30
Male/female (n)	16/4	14/6	0.48
Body weight (kg)	71.4 ± 14.4	69.5 ± 12.2	0.37
BMI	26.6 ± 4.6	25.0 ± 3.1	0.25
Hypertension	14 (70)	12 (60)	0.52
Smoking, ever	6 (30)	5 (25)	0.73
Lipid profile			
LDL (mg/dl)	91 ± 26	84 ± 20	0.44
TG (mg/dl)	152 ± 92	151 ± 81	0.83
HDL (mg/dl)	46 ± 14	40 ± 10	0.17
TG/HDL	3.6 ± 2.1	3.7 ± 2.1	0.73
Glycemic marker			
HbA1c (%)	7.2 ± 0.6	7.4 ± 1.1	0.51
FBS (mg/dl)	144 ± 41	136 ± 24	0.48
HOMA-IR	2.6 ± 1.9	2.5 ± 1.8	0.79
iHOMA 2%S	113 ± 94	134 ± 75	0.52
Keton body (μmol/l)	126 ± 116	120 ± 105	
Adipose-associated marker			
EAT volume (cm^3^)	115 ± 22	108 ± 25	0.38
TNF-α (pg/ml)	2.4 ± 0.7	2.2 ± 0.7	0.78
PAI-1 (ng/ml)	42.2 ± 16.1	45.7 ± 14.6	0.41
BNP (pg/ml)	82 ± 88	102 ± 129	0.62
Alb/Cre ratio (mg/g CRE)	21 ± 39	38 ± 68	0.50

Data are presented as mean ± SD or the number (percentage)

*LDL* low density lipoprotein cholesterol, *HDL* high density lipoprotein cholesterol, *HOMA-IR* homeostatic model assessment insulin resistance, *iHOMA-2* interactive 24-variable model homeostatic model assessment insulin resistance-2, *EAT* epicardial adipose tissue, *TNF-α* tumor necrotic factor-α, *PAI-1* plasminogen activator inhibitor-1, *BNP* brain natriuretic peptide, *Alb/Cre ratio* albumin/creatinine ratio

**Table 2:** Medications before the allocation

	Dapagliflozin	Conventional therapy
α-GI	2 (10)	1 (5)
DPP-4 inhibitor	5 (25)	6 (30)
Biguanide	6 (30)	5 (25)
Sulfonyl urea	2 (10)	1 (5)
Glinide	0 (0)	0 (0)
Pioglitazone	1 (5)	0 (0)

Data are presented as the number (percentage)

*α-GI* α-glucosidase inhibitors, *DPP-4* dipeptidyl peptidase-4

**Table 3:** Newly additional medications after the allocation

	Dapagliflozin (n = 20)	Conventional therapy (n = 20)
SGLT-2 inhibitor	20 (100)	0
α-GI	0 (0)	3 (15)
DPP-4 inhibitor	0 (0)	14 (70)
Biguanide	0 (0)	1 (5)
Sulfonyl urea	0 (0)	1 (5)
Glinide	0 (0)	1 (5)
Pioglitazone	0 (0)	0

Data are presented as the number (percentage)

*SGLT-2* sodium-glucose co-transporter-2, *α-GI* α-glucosidase inhibitors, *DPP-4* dipeptidyl peptidase-4

### Changes in body weight and laboratory data (Table 4)

At baseline, no significant differences were observed in HbA1c, LDL, TG, and HDL levels between the dapagliflozin and conventional treatment groups (7.2 ± 0.6 vs. 7.4 ± 1.1%, p = 0.51; 91 ± 26 vs. 84 ± 20 mg/dL, p = 0.44; 152 ± 92 vs. 151 ± 81 mg/dL, p = 0.83; 46 ± 14 vs. 40 ± 10 mg/dL, p = 0.17, respectively).

**Table 4:** Change rate in each marker after treatment

	Dapagliflozin (n = 20)	Conventional therapy (n = 20)	p value, dapagliflozin vs. conventional
Δ body weight (kg)	− 2.9 ± 3.4**	0.2 ± 2.4	0.01
Lipid profile			
ΔLDL (mg/dl)	6.0 ± 26.5	− 1.0 ± 13.7	0.41
ΔTG (mg/dl)	− 33.4 ± 66.2*	3.2 ± 19.0	0.07
ΔHDL (mg/dl)	5.1 ± 15.2	− 0.7 ± 4.8	0.21
ΔTG/HDL	− 0.8 ± 1.7**	0.2 ± 0.8	0.06
Glycemic marker			
ΔHbA1c (%)	− 0.41 ± 0.21**	− 0.19 ± 0.25	0.22
ΔHOMA-IR	− 0.99 ± 1.90**	− 0.42 ± 0.86*	0.64
ΔiHOMA2%S	65.7 ± 70.9**	42.2 ± 61.1	0.36
ΔBNP (pg/ml)	− 24 ± 83	− 12 ± 54	0.68
ΔAlb/Cre (mg/g·CRE)	− 3.8 ± 29.6	8.7 ± 43.2	0.38

*LDL* low density lipoprotein cholesterol, *HDL* high density lipoprotein cholesterol, *HOMA-IR* homeostatic model assessment insulin resistance, *iHOMA-2* interactive 24-variable model homeostatic model assessment insulin resistance-2, *BNP* brain natriuretic peptide, *Alb/Cre ratio* albumin/creatinine ratio

Data are expressed as mean ± SD; ** p < 0.05 compared with baseline of each group, * p < 0.1 compared with baseline of each group

Compared with the baseline, at the 6-month follow-up, the decreases in body weight in the dapagliflozin group were significantly greater than in the conventional treatment group (− 2.9 ± 3.4 vs. 0.2 ± 2.4 kg, p = 0.01). However, the change in HbA1c level in the dapagliflozin group was comparable with that in the conventional treatment group (− 0.41 ± 0.21 vs. − 0.19 ± 0.25%, p = 0.22). In addition, the change in the TG/HDL ratio in the dapagliflozin group tended to be greater than that in the conventional treatment group (− 0.8 ± 1.7 vs. 0.2 ± 0.8, p = 0.06).

Regarding insulin resistance, the changes in HOMA-IR and iHOMA2 values in both groups were similar, and both groups showed improved insulin resistance compared with both the baselines.

### Changes in adipose-associated markers (Table 5)

Overall EAT volume was 112 ± 24 cm^3^. At baseline, the EAT volumes in the dapagliflozin and conventional treatment groups were 115 ± 22 and 108 ± 25 cm^3^, respectively, and were comparable with each other. At the 6-month follow-up, the EAT volume in the dapagliflozin group decreased significantly at follow-up compared with the baseline. Furthermore, the change in EAT volume in the dapagliflozin group was significantly greater than that in the conventional treatment group (− 16.4 ± 8.3 vs. 4.7 ± 8.8 cm^3^, p = 0.01).

**Table 5:** Change rate of adipose-associated markers after treatment

	Dapagliflozin (n = 20)	Conventional therapy (n = 20)	p value, dapagliflozin vs. conventional
ΔEAT volume (cm^3^)	− 16.4 ± 8.3**	4.7 ± 8.8	0.01
ΔTNF-α (pg/ml)	− 0.5 ± 0.7**	0.03 ± 0.3	0.03
ΔPAI-1 (ng/ml)	− 10.1 ± 18.8*	− 2.0 ± 9.7	0.18

*EAT* epicardial adipose tissue, *TNF-α* tumor necrosis factor-α, *PAI-1* plasminogen activator inhibitor-1

Data are expressed as mean ± SD. ** p < 0.05 compared with baseline of each group, * p < 0.1 compared with baseline of each group

In addition, the serum PAI-1 level tended (non-significantly) to decrease at the 6-month follow-up, from 42.2 ± 16.1 to 32.9 ± 14.4 ng/mL (p = 0.07), in the dapagliflozin group. However, the change in the serum PAI-1 level in the dapagliflozin group was similar to that in the conventional treatment group. On the other hand, the TNF-α level decreased significantly from 2.4 ± 0.7 to 1.9 ± 0.5 pg/mL (p = 0.04) in the dapagliflozin group. In addition, the change in the TNF-α level in the dapagliflozin group was significantly greater than that in the conventional treatment group (− 0.5 ± 0.7 vs. 0.03 ± 0.3 pg/ml, p = 0.03).

**Fig. 2:**
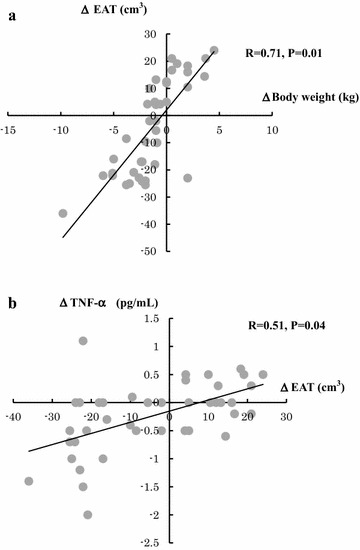
The correlation between the change in EAT volume and those in body weight or inflammatory marker (TNF-α). **a** A positive significant correlation was observed between the changes in EAT volume and body weight. **b** A positive significant correlation was observed between the changes in EAT volume and TNF-α level

Furthermore, the changes in the EAT volume and body weight showed significantly positive correlation (r = 0.71, p = 0.01) as did the changes in the EAT volume and TNF-α level (r = 0.51, p=0.04) (Fig. 2).

## Discussion

The main findings of the present study are as follows: (1) Dapagliflozin significantly improved not only glycemic control but also EAT volume, and (2) The significant correlation between change in EAT volume and those in body weight or TNF-α level were observed. Thus, dapagliflozin improved not only glycemic control but also levels of systemic inflammation markers.

### SGLT-2 inhibitor, EAT and inflammation

Obesity interacts with DM closely and is associated with cardiovascular complications [2]. Adipose tissue has a high capacity to secrete many pro-inflammatory substances including PAI-1 and TNF-α [3]. Systemic micro-inflammation causes insulin resistance and is located in the central of the initiation and the propagation of atherosclerosis [17]. Furthermore, excessive adipose tissue increases the infiltration of pro-inflammatory immune cells into metabolic tissues and causes phenotypic shifts in macrophages [18, 19]. In fact, overweight people with enhanced accumulation of visceral fat have increased serum levels of pro-inflammatory cytokines such as TNF-α and PAI-1 [4–6]. Several reports described that weight loss through diet and exercise therapy in obese patients significantly increased adiponectin and decreased PAI-1 levels [20, 21]. Glucosuria induced by SGLT-2 inhibitors is also typically associated with a net loss of ~ 200–300 kcal, leading to weight reductions of ~ 2–3 kg over 24–52 weeks. Furthermore, previous studies also reported that treatment with an SGLT-2 inhibitor even improved the inflammatory state [10, 22]. In addition, significant weight loss in obese patients has been associated with noteworthy reduction in the EAT volume [23, 24]. Taken together, the SGLT-2 inhibitor treatment in the present study might support an improved inflammatory state via a decrease in fat accumulation presumably due to loss of body weight, because changes in the EAT volume and body weight or inflammatory marker levels (TNF-α) were significantly correlated. In fact, recent studies have reported the effect of dapagliflozin, ipragliflozin, and luseogliflozin treatment on changes in body weight, EAT volume, and inflammatory marker levels [25–27], the results of which are consistent with those of the present study.

Although adiponectin was not measured in the present study, the decreased PAI-1 and TNF-α levels seen might be associated with the improvement in adipocyte function, finally leading to changes in the HbA1c level and iHOMA2%S.

However, a previous report has also described that an SGLT-2 inhibitor reduced fat accumulation (i.e., fatty liver) without changing body weight [28]. In addition, a previous report described that brown adipose tissue weights were lower in a high-fat-diet plus high-dose empagliflozin group than in a high-fat-diet plus low-dose empagliflozin group [22]. Therefore, further studies are warranted to clarify the mechanism of SGLT-2 inhibitor-mediated improvement in the inflammatory state and the ectopic fat.

### SGLT-2 inhibitor treatment and protection against cardiovascular events

Several major trials have reported that treatment with an SGLT-2 inhibitor reduced the occurrence of cardiovascular events [13, 29]. The potential mechanisms through which these events were reduced by an SGLT-2 inhibitor include: improvement in glucose perturbations and insulin sensitivity; reduction in blood pressure and arterial stiffness; reduction in body fat and fat mass; reduction in the levels of uric acid; positive effects on proteinuria and kidney function; and positive effects on lipid parameters [30]. The present study did not evaluate blood pressure, arterial stiffness, or uric acid levels. However, glycemic parameters such as the HbA1c level and iHOMA2%S were improved by treatment with dapagliflozin. Interestingly, the LDL level increased slightly compared with the baseline in patients treated with dapagliflozin. However, a previous report indicated that, although an SGLT-2 inhibitor slightly increased the overall LDL level, it also significantly reduced small dense LDLs [31]. An increase in small dense LDLs was associated with the occurrence of myocardial infarction [32]. Though the present study did not evaluate the small dense LDL level, this level correlates with the TG/HDL ratio [33]. Thus, the decrease in the TG/HDL ratio seen in the present study probably indicates a reduction in the small dense LDL level.

As above-mentioned, the treatment by SGLT-2 inhibitors lead to weight reductions of ~ 2–3 kg over 24–52 weeks. Recently, specific genetic manipulations in adipose tissue and administration of pioglitazone, sitagliptin and metformin, or glucagon-like peptide 1 receptor agonist have also improved EAT volume or the inflammatory state [24, 34–36]. However, a previous report described that diet (calorie restriction) was the most effective therapy to reduce EAT volume, compared with surgery and medications [37]. Therefore, calorie loss through glucosuria by SGLT-2 inhibitor treatment might contribute to the reduction in EAT volume in the present study. The increase in glucagon by SGLT-2 inhibitors was also associated with a decrease in fat accumulation [38].

Visceral adiposity is associated with an increased risk of type 2 DM, cardiovascular complications, and overall mortality, primarily related to abnormal adipocyte biology. There is also altered production of adipocytokines, leading to modulation of cardiovascular pathways that could promote atherosclerosis [39, 40]. EAT is an atherogenetic property and reflects visceral adiposity. Interestingly, EAT thickness is significantly higher in patients with type 2 DM [41]. In general, EAT-derived bioactive molecules, including inflammatory and oxidative stress mediators, are pathophysiological candidates for atherogenesis [42]. In addition, epicardial and pericardial fat volumes are both strongly correlated with the number of atherosclerotic plaques [8]. In fact, the inflammatory status of EAT in obese patients who were candidates for coronary artery bypass graft was more severe than that of subcutaneous fat located in the legs [43]. Furthermore, EAT volume itself was associated with an increased risk of a cardiovascular event [44]. Taken together, though various mechanisms to decrease cardiovascular events have been reported, the present study indicates that the decrease in EAT volume itself might also directly contribute to plaque stability and finally a reduction in the occurrence of myocardial infarction.

## Limitations

The limitations of this study are as follows. First, the number of study patients was small, although this is a prospective study. Second, increased adiponectin values have been reported to possess an anti-atherosclerotic effect [45, 46]. However, in the present study, adiponectin levels were not measured. Third, some antidiabetic medications such as sitagliptin and metformin, and pioglitazone have been reported to be effective in the reduction of EAT volume or improvement in the inflammatory state [34, 35]. Therefore, these medications could be confounding factors in the result of the present study. In addition, treatment with an SGLT-2 inhibitor has been reported to reduce skeletal muscle mass [25, 47]. However, in the present study, we did not measure the skeletal muscle mass. Furthermore, the reduction of EAT volume might also be achieved with weight loss alone. Therefore, this effect might not be specific to dapagliflozin. Fourth, the present study evaluated the change in EAT volume. However, we did not evaluate the relationship between changes in EAT and those of plaque composition and volume. Finally, the observational period in the present study was only 6 months. Therefore, no definite conclusion can be drawn about long-term effects from the results of the present study. These limitations warrant future studies involving larger populations and longer longitudinal follow-up periods.

## Conclusion

Dapagliflozin might influence not only the improvement in systemic metabolic parameters, including PAI-1 and TNF-α levels, but also the decrease in EAT volume. Thus, DM patients treated with dapagliflozin may have a reduced risk of cardiovascular events.

## Abbreviations

SGLT-2: sodium–glucose co-transporter-2
EAT: epicardial adipose tissue
PAI-1: plasminogen activator inhibitor-1
TNF-α: tumor necrosis factor-α
DM: diabetes mellitus
CAD: coronary artery disease
CT: computed tomography
TG: triglyceride
HDL: high-density lipoprotein cholesterol
LDL: low-density lipoprotein
BNP: brain natriuretic peptide
